# Iron Absorption from Iron-Biofortified Sweetpotato Is Higher Than Regular Sweetpotato in Malawian Women while Iron Absorption from Regular and Iron-Biofortified Potatoes Is High in Peruvian Women

**DOI:** 10.1093/jn/nxaa267

**Published:** 2020-11-13

**Authors:** Roelinda Jongstra, Martin N Mwangi, Gabriela Burgos, Christophe Zeder, Jan W Low, Glory Mzembe, Reyna Liria, Mary Penny, Maria I Andrade, Susan Fairweather-Tait, Thomas Zum Felde, Hugo Campos, Kamija S Phiri, Michael B Zimmermann, Rita Wegmüller

**Affiliations:** ETH Zürich, Laboratory of Human Nutrition, Institute of Food, Nutrition, and Health, Department of Health Sciences and Technology, Zurich, Switzerland; Training and Research Unit of Excellence (TRUE), College of Medicine, University of Malawi, Blantyre, Malawi; Genetics, Genomics, and Crop Improvement Program, International Potato Center, Lima, Peru; ETH Zürich, Laboratory of Human Nutrition, Institute of Food, Nutrition, and Health, Department of Health Sciences and Technology, Zurich, Switzerland; International Potato Center, Nairobi, Kenya; Training and Research Unit of Excellence (TRUE), College of Medicine, University of Malawi, Blantyre, Malawi; Instituto de Investigación Nutricional, Lima, Peru; Instituto de Investigación Nutricional, Lima, Peru; International Potato Center, Maputo, Mozambique; Norwich Medical School, University of East Anglia, Norwich, United Kingdom; Genetics, Genomics, and Crop Improvement Program, International Potato Center, Lima, Peru; Genetics, Genomics, and Crop Improvement Program, International Potato Center, Lima, Peru; Training and Research Unit of Excellence (TRUE), College of Medicine, University of Malawi, Blantyre, Malawi; ETH Zürich, Laboratory of Human Nutrition, Institute of Food, Nutrition, and Health, Department of Health Sciences and Technology, Zurich, Switzerland; ETH Zürich, Laboratory of Human Nutrition, Institute of Food, Nutrition, and Health, Department of Health Sciences and Technology, Zurich, Switzerland; GroundWork, Fläsch, Switzerland

**Keywords:** iron absorption, biofortification, stable isotopes, orange-fleshed sweetpotato, potato

## Abstract

**Background:**

Sweetpotato and potato are fast-maturing staple crops and widely consumed in low- and middle-income countries. Conventional breeding to biofortify these crops with iron could improve iron intakes. To our knowledge, iron absorption from sweetpotato and potato has not been assessed.

**Objective:**

The aim was to assess iron absorption from regular and iron-biofortified orange-fleshed sweetpotato in Malawi and yellow-fleshed potato and iron-biofortified purple-fleshed potato in Peru.

**Methods:**

We conducted 2 randomized, multiple-meal studies in generally healthy, iron-depleted women of reproductive age. Malawian women (*n* = 24) received 400 g regular or biofortified sweetpotato test meals and Peruvian women (*n* = 35) received 500 g regular or biofortified potato test meals. Women consumed the meals at breakfast for 2 wk and were then crossed over to the other variety. We labeled the test meals with ^57^Fe or ^58^Fe and measured cumulative erythrocyte incorporation of the labels 14 d after completion of each test-meal sequence to calculate iron absorption. Iron absorption was compared by paired-sample *t* tests.

**Results:**

The regular and biofortified orange-fleshed sweetpotato test meals contained 0.55 and 0.97 mg Fe/100 g. Geometric mean (95% CI) fractional iron absorption (FIA) was 5.82% (3.79%, 8.95%) and 6.02% (4.51%, 8.05%), respectively (*P* = 0.81), resulting in 1.9-fold higher total iron absorption (TIA) from biofortified sweetpotato (*P* < 0.001). The regular and biofortified potato test meals contained 0.33 and 0.69 mg Fe/100 g. FIA was 28.4% (23.5%, 34.2%) from the regular yellow-fleshed and 13.3% (10.6%, 16.6%) from the biofortified purple-fleshed potato meals, respectively (*P* < 0.001), resulting in no significant difference in TIA (*P* = 0.88).

**Conclusions:**

FIA from regular yellow-fleshed potato was remarkably high, at 28%. Iron absorbed from both potato test meals covered 33% of the daily absorbed iron requirement for women of reproductive age, while the biofortified orange-fleshed sweetpotato test meal covered 18% of this requirement. High polyphenol concentrations were likely the major inhibitors of iron absorption. These trials were registered at www.clinicaltrials.gov as NCT03840031 (Malawi) and NCT04216030 (Peru).

See corresponding commentary on page 3051.

## Introduction

Iron deficiency anemia (IDA) is a major cause of the global burden of disease in women (1). It causes fatigue and reduces physical performance, and during pregnancy increases the risk of preterm birth and low birth weight (2, 3). In low- and middle-income countries (LMICs), a common cause of iron deficiency (ID)/IDA is insufficient intake of bioavailable iron from monotonous plant-based diets (4). The estimated prevalence of ID in women of reproductive age is 15.1% in Malawi (5), while anemia prevalence in this population group is 21.1% in Peru (6). A common strategy to combat ID is iron supplementation, but compliance with iron supplementation is often low, and high iron doses may increase infection risk and cause gut dysbiosis and inflammation (7). Thus, alternative approaches to increase iron intakes in women in LMICs are needed (8).

Biofortification, the process by which the micronutrient quantity of food crops is enhanced through conventional plant breeding or biotechnology, is a promising approach to increase iron content in staple food crops (9). Efficacy trials of iron-biofortified beans and pearl millet have shown these crops can improve iron status, increasing serum ferritin and total body iron (10, 11).

Sweetpotato breeding efforts during the past decade in sub-Saharan Africa have focused on developing vitamin A–rich, orange-fleshed sweetpotato varieties to address the high prevalence of vitamin A deficiency (48%) among young children (12). Sweetpotato was one of the most harvested food crops in Malawi in 2018 (13, 14) and orange-fleshed types make up a significant percentage of total sweetpotato production. Potato is the third most harvested food crop in the world and Peru is the biggest producer in Latin America (13), with Peruvian women consuming up to 800 g of potato daily (15). In Malawi, data on sweetpotato consumption in women are not available, but >60% of children consumed any type of sweetpotato at least once a week (16) and 100 g of available orange-fleshed varieties meets the daily vitamin A needs of young children (12). Thus, these countries are potential targets for iron biofortification of these crops. For both crops, there has been considerable progress in increasing their iron content through conventional breeding (17–19).

Iron absorption from potato and sweetpotato is predicted to be high due to the relatively low phytate and high ascorbic acid content in comparison to cereals (20–23). In vitro iron bioaccessibility (amount of iron released from the food matrix during in vitro gastrointestinal digestion and available for absorption at the intestinal level) was 63–79% in potato (24) and 47–62% in sweetpotato (21) compared with 6–24% in pearl millet, different types of beans, and rice (25).

The aims of our study were to assess iron bioavailability from test meals prepared with*1)* regular (a released orange-fleshed variety) and iron-biofortified orange-fleshed sweetpotato (a clone from the on-going breeding program for high iron) in Malawian women and *2)* from regular yellow-fleshed potato (a commercial popular landrace) and iron-biofortified purple-fleshed potato (a clone from the on-going breeding program for high iron) in Peruvian women. In both studies, we assessed iron bioavailability by measuring iron stable isotope incorporation into erythrocytes after consumption of multiple labeled test meals (11, 26).

## Methods

### Study site and subjects

The sweetpotato study was conducted from March to April 2019 in the Zomba area (altitude, 949 m) of southern Malawi. The potato study was conducted from May to August 2019 in the department of Huancavelica (altitude, 3600 m) in the central highlands of Peru.

**FIGURE 1:**
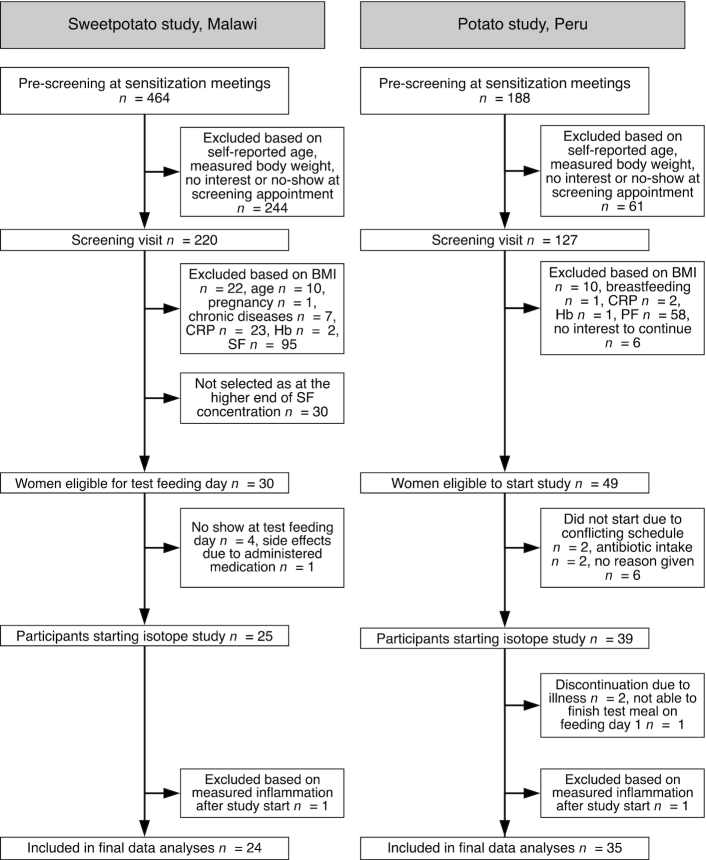
Study overview diagrams for the sweetpotato study in Malawi and the potato study in Peru. CRP, C-reactive protein; Hb, hemoglobin; PF, plasma ferritin; SF, serum ferritin.

Both studies used a 2-stage screening approach (**Figure 1**). In Malawi, we recruited women in communities surrounding Zomba Central Hospital. In Peru, we recruited women at the National University and the Instituto Pedagógico of Huancavelica. We first prescreened interested women for the inclusion criteria of age (self-reported) and weight (measured in both studies using a SECA scale; model 813; SECA). Women who met these inclusion criteria were then invited for full screening. The inclusion criteria for both studies were as follows: body weight (<65 kg), BMI (kg/m^2^; 18.5–25.0), age 18–35 y (Malawi) and 18–25 y (Peru), serum ferritin (SF; Malawi) or plasma ferritin (PF; Peru) concentration ≤25 µg/L, C-reactive protein (CRP) concentration <5 mg/L and hemoglobin (Hb) >80 g/L [adjusted for altitude (27) in Peru], not pregnant (assessed by using a urine pregnancy test) or lactating, no chronic diseases or medications that could influence iron metabolism, no current intake of mineral and/or vitamin supplements or willing to discontinue supplementation 2 wk prior to study start, no current participation in any clinical trial or during the past month prior to study start, able to comply with all study procedures, and no presence of fever (>37.5°C) on the first study day. For Malawi, additional exclusion criteria were: having taken an iron supplement in the last 3 mo, enrollment in any supplemental food program, and self-reported alcohol consumption of >2 drinks/d.

### Ethical issues

The sweetpotato study in Malawi was approved by the Ethics Commission of ETH Zürich, Switzerland, and by the College of Medicine Research and Ethics Committee, Malawi. The potato study in Peru was approved by the Comité de Ética en Investigación of Instituto de Investigación Nutricional and received written permission without full review from the Ethics Commission of ETH Zürich. In both studies, participants gave written informed consent. The studies were registered under Clinical Trial Identifier numbers NCT03840031 (Malawi) and NCT04216030 (Peru).

### Intervention

**FIGURE 2:**
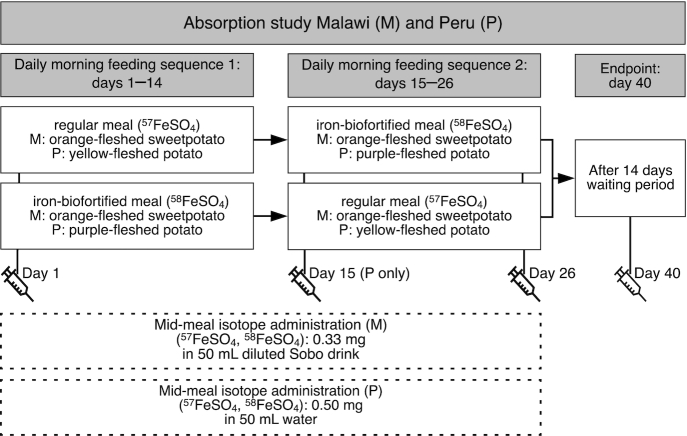
Study design for the sweetpotato study in Malawi (M) and the potato study in Peru (P). In both studies women were randomly assigned to either the regular OFSP (M), YFP (P), or iron-biofortified OFSP (M), PFP (P) test meals with iron isotopic administration (regular test meal, ^57^Fe, or iron-biofortified test meal, ^58^Fe) for 10 consecutive days, after which they crossed over to the other test meal sequence. OFSP, orange-fleshed sweetpotato; PFP, purple-fleshed potato; Sobo® (Southern Bottlers Ltd); YFP, yellow-fleshed potato.

In Malawi, we invited the 30 women with the lowest SF results at screening, and who met all the other inclusion criteria, for a test feeding day. On this day, we fed the women a standardized test meal consisting of 400 g mashed orange-fleshed sweetpotato (regular or iron-biofortified). After finishing the test meal, women remained fasting for 3 h. Information on sociodemographic status was collected via a standardized electronic questionnaire using the REDCap^®^ platform (Vanderbilt) (28). At the end of the 3-h post–test meal period, we administered a single 400-mg dose of albendazole and the first dose of malaria prophylaxis (the dose was 3 tablets for study participants <60 kg or 4 tablets for study participants ≥60 kg; each tablet contained 40 mg dihydroartemisinin and 320 mg piperaquine). Women were instructed to take the next 2 doses after lunch the next day and the day after. One woman experienced a mild adverse drug reaction and was excluded. Four women did not show up on the test feeding day. Therefore, 25 women started the iron absorption study 3 d after the test feeding day. In Peru, we did not have a test feeding day, and all women began the absorption study 1 wk after screening. We used a blockwise inclusion of a maximum of 10 women/wk until 39 women were enrolled, which was reached after 8 wk. In both studies, we used a nonblinded, randomized, crossover, multiple-meal design to measure fractional iron absorption (FIA) and total iron absorption (TIA) from *1*) regular and iron-biofortified orange-fleshed sweetpotato meals in Malawi and *2*) regular yellow-fleshed and iron-biofortified purple-fleshed potato meals in Peru (**Figure 2**).

In both studies on the first study day, we collected a venous blood sample to determine baseline isotopic composition as well as iron and inflammation status markers. Women began consuming the first randomly assigned test meal type labeled with the corresponding iron isotope (in Malawi: 0.33 mg ^57^Fe or ^58^Fe for the regular and biofortified sweetpotato; in Peru: 0.5 mg ^57^Fe or ^58^Fe for the regular and biofortified potato) (Figure 2). The assigned test meal was consumed as breakfast for 10 consecutive days, interrupted by a weekend break. On day 15, all participants switched over to the second test meal type, which they consumed for another 10 d. Each test meal was weighed before and after consumption to calculate the exact intrinsic iron intake. Meals were consumed under direct supervision by the study team after overnight fasting was confirmed. We observed the women for 3 h after the test meals and no food or drink was allowed other than mineral water (500 mL). On day 15 (Peru only), 26, and 40, trained staff collected venous blood to measure iron and inflammation markers, and isotopic composition was measured in the day 26 and 40 samples. In Malawi, a medical doctor assessed participants who tested positive for malaria or who presented with other health problems and decided on further treatment. In Peru, subjects developing a fever or other symptoms of illness were discontinued from the study and advised to consult their medical doctor.

### Test meal preparation and composition

In Malawi, the test meals consisted of a standardized recipe including 400 g of steamed and mashed orange-fleshed sweetpotato (details of meal preparation are provided in the **Supplemental Material**) of either the released variety Irene (regular, 7.7 ppm Fe in raw roots) or the breeding clone MUSG15052-2 (high-iron, 15.8 ppm Fe in raw roots). Both sweetpotato varieties were bred, grown, and harvested by the International Potato Center (CIP) in Mozambique in collaboration with the Instituto de Investigação Agrária de Mozambique. We served 250 mL diluted Sobo® Squash Orange (Southern Bottlers Ltd; 1:4 dilution using bottled water) with every test meal, and an additional 50 mL was used for isotope administration after half of the test meal was consumed.

In Peru, the test meals consisted of a standardized meal of 500 g of boiled and mashed potato (details of meal preparation are provided in the Supplemental Material) of either the released variety Peruanita (regular yellow-fleshed potato, 3.0 ppm Fe in raw tubers) or the breeding clone CIP306417.79 (high-iron purple-fleshed potato, 5.6 ppm Fe in raw tubers). The regular yellow-fleshed variety was purchased from a local farmer in Paucartambo, while the iron-biofortified purple-fleshed clone was grown and harvested by CIP in the same locality. Each participant received 200 mL of mineral water with every test meal and an additional 50 mL was used for isotope administration after half of the test meal was consumed.

### Stable isotope labels

^57^FeSO_4_ and ^58^FeSO_4_ were prepared from ^57^Fe-enriched elemental iron (95.6% isotopic enrichment) and ^58^Fe-enriched elemental iron (99.9% isotopic enrichment; all Chemgas, Boulogne, France) by dissolution in 0.1 M sulfuric acid. Individual 5-mL doses [containing 3.3 mg (Malawi) and 5.0 mg (Peru) of labeled iron], sufficient to label the 10 test meals for each participant and meal type were prepared. These were stored in 5-mL Teflon vials and flushed with argon to keep the iron in the +II oxidation state. All labeled iron compounds were analyzed for iron isotopic composition and iron concentration by inductively coupled plasma (ICP)–MS (Neptune; Thermo-Fisher Scientific).

### Test meal analyses

We analyzed the nutrient composition of both fresh uncooked varieties of sweetpotato/potato as well as their test meals. Iron and calcium concentrations were determined by ICP–MS (iCAP; Thermo-Fisher Scientific) after mineralization using HNO_3_ by microwave digestion (MLS TurboWave; MLS GmbH). Phytic acid (PA) concentration was measured using a modification of the Makower method (29), as described previously by our laboratory (30). We measured total polyphenol (PP) concentration using a modified Folin-Ciocalteau method (31, 32), and expressed the results as Gallic acid equivalents (GAEs). For all Malawi samples, ascorbic acid (AA) was analyzed after stabilization and extraction in meta-phosphoric acid and reduction via dithiothreitol with HPLC (Acquity H-class UPLC system, 4824949; Waters AG). The Peru samples were analyzed for AA by UV/Visible spectroscopy (UV 160A; Shimadzu Corp), as described by Burgos et al. (33).

### Blood collection and analysis

In Malawi, Hb and CRP concentrations were measured using a QuikRead device (QuikRead go CRP+Hb; Orion Diagnostica) and the presence of *Plasmodiumfalciparum* malaria antigen using a rapid immunochromatographic test (Parahit^®^*f* ver 1.0; M/S Span Diagnostics Inc). One serum aliquot from each screened participant was sent to Blantyre, Malawi, for SF analyses at the Blantyre Diagnostics Laboratory using a manual ELISA microplate immunoenzymometric assay (BXE0891A; Biorex Diagnostics). In Peru, Hb was measured in whole blood using a 5-differential flow cytometer (Nihon Kohden), PF by electrochemiluminescence (Cobas E 411; Roche Diagnostics), and CRP by turbidimetry (Vitros 4600; Ortho Clinical Diagnostics). In Malawi, the presence of *P. falciparum* malaria antigen as described for the screening and Hb concentration using a hematology analyzer (Xp-300; Sysmex Suisse AG) were assessed at all blood collection points during the feeding days to check participants’ health status. In Peru, Hb was measured in whole blood as described for the screening samples at all blood collection points during the feeding days.

All aliquoted samples collected during the absorption studies were stored frozen at −18°C and shipped on dry ice from both study sites to the Human Nutrition Laboratory of ETH Zürich. Analyses of ferritin, soluble transferrin receptor (sTfR), CRP, and α1-acid glycoprotein (AGP) were done using immunoassay (34). Whole-blood samples were analyzed in duplicate for their iron isotopic composition after mineralization by microwave digestion (MLS TurboWave; MLS GmbH) followed by iron separation from the blood matrix by anion-exchange chromatography and a subsequent purification step by precipitation as iron hydroxide using ICP-MS (Neptune; Thermo-Fisher Scientific) (35).

### Calculation of iron absorption

We calculated the amounts of ^57^Fe and ^58^Fe isotopic labels in RBCs 14 d after each meal sequence on the basis of the shift in iron-isotope ratios and the estimated amount of iron circulating in the body. Circulating iron was calculated based on blood volume estimated from height and weight and mean Hb concentration at days 1 and 40 (36). The calculations were based on the principles of isotope dilution (37). For calculation of fractional absorption, it was assumed that 80% of the absorbed iron was incorporated into RBCs (38).

### Sample size calculation

Both studies assumed an SD of 0.18 on log-transformed FIA from a previous study using iron-biofortified millet in nonpregnant Beninese women (11). With the type I error rate set to 5% we had 90% power to detect a 35% difference in TIA (regular vs. iron-biofortified test meal) with a sample size of 20 participants. To account for potential dropouts, which were anticipated to be higher in Peru, we enrolled 25 women in Malawi and 39 women in Peru.

### Statistical analysis

We performed statistical analyses using STATA (version 15.0; StataCorp LLC) and Excel (version 2018; Microsoft). Normally distributed data tested by Shapiro-Wilk were reported as means (±SDs) or geometric means (±SDs) if normally distributed after log transformation. Non–normally distributed data after log transformation were reported as medians (IQRs). To compare FIA within participants from the 2 test meals (regular vs. iron-biofortified), we used paired *t* test. In order to assess within-subject variation in iron status and inflammation on measured time points, ANOVA or Wilcoxon signed-rank test was performed. *P* values <0.05 were considered statistically significant.

## Results

In Malawi, 1 woman had to be excluded from the study due to an elevated CRP concentration (>5 mg/L) and the presence of fever on day 1 (Figure 1). Two women were treated during the test meal feedings with antibiotics and 1 woman was treated for malaria on the final study day. In Peru, 2 women were excluded on day 1 (1 woman with a CRP concentration >5 mg/L, 1 woman not able to consume the whole test meal) and 2 women dropped out due to illness during the feeding period (Figure 1).

The composition of the sweetpotato and potato test meals is summarized in **Table 1**. In Malawi, the average daily consumption (±SD) of the regular and iron-biofortified orange-fleshed sweetpotato meals was 433 ± 5 g and 444 ± 5 g, respectively, resulting in a daily intrinsic iron intake of 2.38 (± 0.03) mg from the regular test meal and 4.32 (± 0.04) from the iron-biofortified test meal. PA concentrations were low in both test meals, while AA content was low (1.1 ± 0.2 mg/100 g) in the biofortified meal and moderate (7.4 ± 0.2 mg/100 g) in the regular meal. PP content, expressed as GAEs, was high, with 198 and 255 mg PP in the total regular and biofortified meals, respectively. Sobo^®^ (Southern Bottlers Ltd), which was used to administer the isotopes in Malawi, contributed only 0.48 mg AA per meal serving. In Peru, the average daily consumption (±SD) of the regular and iron-biofortified potato meals was 500 ± 1 g and 500 ± 0.5 g, respectively, resulting in a daily intrinsic iron intake of 1.63 ± 0.02 mg from the regular yellow-fleshed potato meal and 3.47 ± 0.05 mg from the biofortified purple-fleshed potato meal. Both test meals contained some PA and AA and high concentrations of PP, being 3.5-fold higher in the biofortified purple-fleshed meal compared with the regular yellow-fleshed meal (528 and 148 mg GAEs, respectively).

**TABLE 1:** Composition of the test meals provided to women in Malawi and Peru^1^

	Sweetpotato (Malawi)		Potato (Peru)	
Test meal type	Regular (orange-fleshed)	Iron-biofortified (orange-fleshed)	Regular (yellow-fleshed)	Iron-biofortified (purple-fleshed)
Variety name	Irene	Clone MUSG-15052-2	Peruanita	Clone 306417.79
Iron, native, mg/100 g	0.55 ± 0.03	0.97 ± 0.07	0.33 ± 0.03	0.69 ± 0.07
AA, mg/100 g	7.4 ± 0.1	1.1 ± 0.2	8.8 ± 2.5	12.7 ± 1.3
AA:iron, molar ratio	3.7	0.3	6.5	5.1
PA, mg/100 g	<LOQ^2^	<LOQ^2^	4.8 ± 0.9	31.7 ± 5.1
PA:iron, molar ratio	—	—	1.0	3.4
Total polyphenols, mg GAE/100 g	45.8 ± 3.9	57.4 ± 2.8	29.6 ± 2.3	105.6 ± 7.9
Calcium, mg/100g	26.3 ± 0.3	40.3 ± 0.6	5.4 ± 0.6	7.3 ± 0.5

1 Values are means ± SDs unless otherwise indicated. AA, ascorbic acid; GAE, gallic acid equivalents; LOQ, limit of quantification; PA, phytic acid.

2 Below the LOQ of 12 mg/100 g dry matter.

Baseline characteristics for the Malawian and Peruvian women are shown in **Table 2**. In Malawi, 5 women (20.8%) were ID (PF ≤12 µg/L) and 4 of these women had IDA; an additional 5 women were anemic without ID. In Malawi, due to a technical error in the serum ferritin measurements during screening, only 14 of the 24 women had a PF ≤25 µg/L at baseline (PF range: 2.69–115.40 µg/L). In Peru, 31 of the 34 women had a PF ≤25 µg/L at baseline, 22 were ID (64.7%) and 12 of these women were IDA, while 2 women were anemic without ID. Iron and inflammation status markers did not significantly change over the 6-wk study period in both studies.

**TABLE 2:** Baseline (day 1) characteristics of women who consumed the test meals and were included for final data analysis in Malawi (*n* = 24) and Peru (*n* = 35)^1^

	Sweetpotato study (Malawi)	Potato study (Peru)
Age, y	24.9 ± 4.81	21.4 ± 2.0
Weight, kg	54.7 ± 4.44	51.9 ± 6.4
BMI, kg/m^2^	21.8 ± 2.0	22.5 ± 1.9
Hemoglobin, g/L	123.1 ± 11.2	122.5 ± 9.3^2^
Plasma ferritin, µg/L	21.4 [15.0, 30.5]	9.6 [7.6, 12.3]
Soluble transferrin receptor, mg/L	6.4 (5.5–7.7)	5.7 (5.1–7.3)
C-reactive protein, mg/L	0.53 [0.35, 0.82]	0.11 [0.07, 0.20]
α1-Acid glycoprotein, g/L	0.58 (0.43–0.66)	0.42 (0.36–0.51)

1 Values are means ± SDs, geometric means [95% CI], or medians (IQR).

2 Hemoglobin values for Peru were corrected for altitude (27).

**FIGURE 3:**
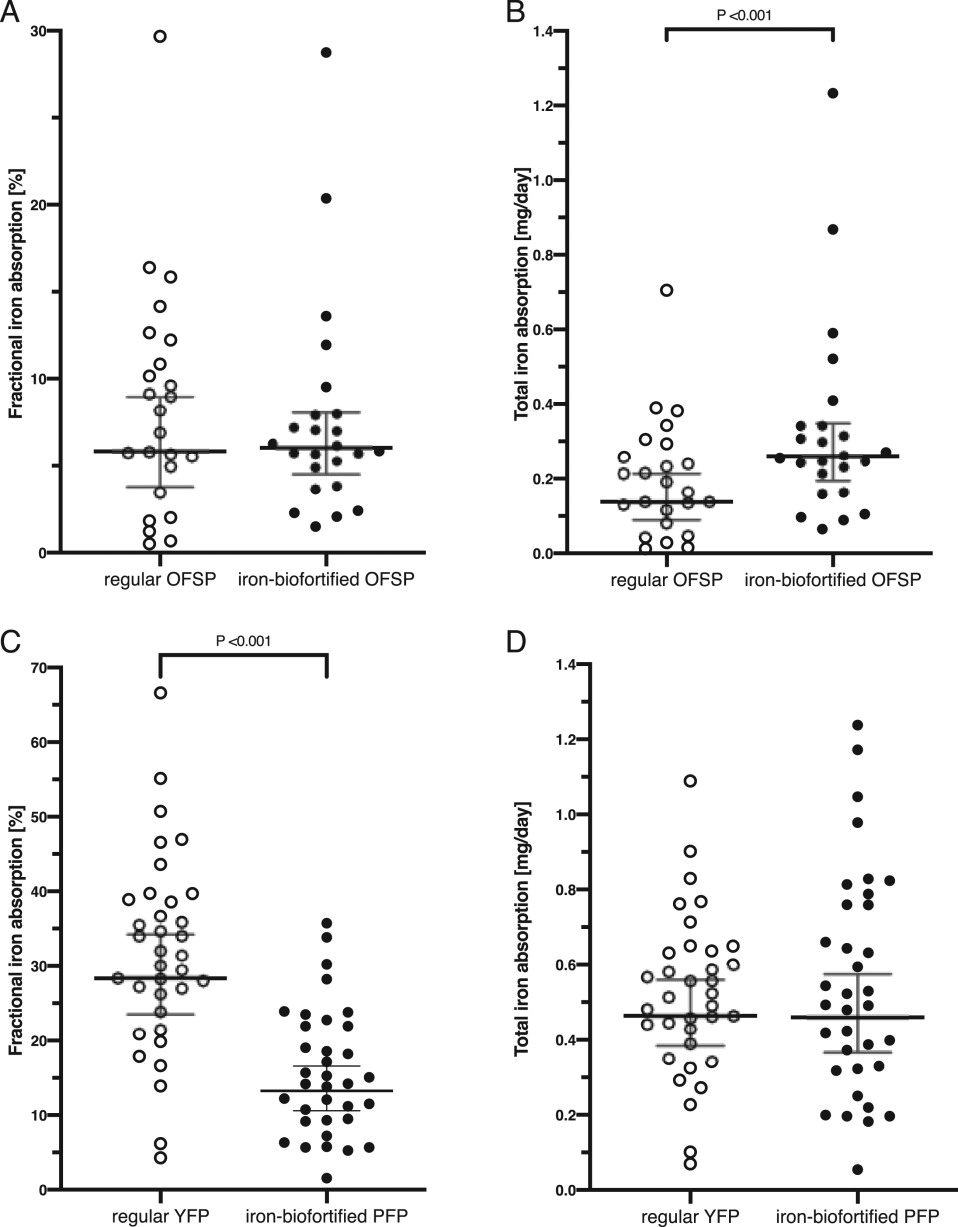
Fractional iron absorption (%) (A, C) and total iron absorption (mg/d) (B, D) from regular and iron-biofortified OFSP test meals in Malawian women (A, B; *n* = 24) and from regular YFP and iron-biofortified PFP test meals in Peruvian women (C, D; *n* = 35). Isotopic administration in both studies: ^57^Fe for the regular test meal and ^58^Fe for the iron-biofortified test meal. The horizontal lines represent geometric means (95% CIs). OFSP, orange-fleshed sweetpotato; PFP, purple-fleshed potato; YFP, yellow-fleshed potato.

In Malawi, FIA (geometric mean; 95% CI) did not differ (*P* = 0.81) between the regular (5.82; 3.79, 8.95) and iron-biofortified sweetpotato test meals (6.02; 4.51, 8.05) (**Figure 3**). TIA in milligrams per day (geometric mean; 95% CI) was significantly higher (*P* < 0.001) from the biofortified test meal (0.26; 0.19, 0.35) compared with the regular test meal (0.14; 0.09, 0.21), resulting in a 1.9-fold higher amount of iron absorbed daily and covering 18% of the median daily requirement for absorbed iron in women of reproductive age (39). The geometric mean (95% CI) FIA from the iron-biofortified orange-fleshed sweetpotato test meal for participants with PF >25 µg/L (*n* = 10) was 3.98 (2.49, 6.39) but was 8.09 (5.87, 11.15) for participants with PF ≤25 µg/L (*n* = 14), demonstrating the well-known influence of iron status on FIA. Thus, women with low iron stores would obtain 25% of their daily needs for absorbed iron when consuming the biofortified orange-fleshed sweetpotato test meal.

In Peru, FIA (geometric mean; 95% CI) was significantly higher (*P* < 0.001) from the regular yellow-fleshed potato meal (28.4; 23.5, 34.2) compared with the iron-biofortified purple-fleshed test meal (13.3; 10.6, 16.6) (Figure 3). TIA in milligrams per day (geometric mean; 95% CI), however, was not different (*P* = 0.88) between the regular (0.46; 0.38, 0.56) and biofortified (0.46; 0.37, 0.57) test meal providing 33% of the median daily iron requirement for absorbed iron in women of reproductive age (39).

## Discussion

In Malawi, women absorbed 0.26 mg Fe/d from iron-biofortified orange-fleshed sweetpotato, 0.12 mg more than from regular meals, while FIA did not differ (6%). In contrast, in Peru, the FIA from iron-biofortified purple-fleshed potato (13.3%) was half that from the regular yellow-fleshed variety (28.4%), resulting in the same daily quantity of iron absorbed (0.46 mg). The FIA of both the regular yellow-fleshed and biofortified purple-fleshed potato was above the expected 5–10% for plant-based diets in LMICs (40) when consumed as a single-food test meal. These iron absorption estimates, however, are based on a mixed diet high in PA, the main inhibitor of nonheme iron absorption (41). The inhibitory PA:Fe molar ratio >1 for iron absorption from legume-based meals was only exceeded in the potato test meals; therefore, it is unlikely to have contributed to the low FIA from the orange-fleshed sweetpotato test meals (42). In biofortified beans, the removal of PA only slightly increased iron absorption, suggesting a stronger inhibitory effect of polyphenols (43, 44).

All sweetpotato and potato test meals contained significant amounts of PP (expressed in GAEs), with the biofortified purple-fleshed potato meal containing 3-fold more PP (528 mg) than the regular yellow-fleshed test meal (148 mg) and about twice that of the regular (198 mg) and biofortified (255 mg) orange-fleshed sweetpotato test meals. For sweetpotato, but not potato, PP values are considerably higher than previously reported values of 5–21 mg PP GAEs per 100 g raw orange-fleshed sweetpotato roots (21, 45). The considerably lower FIA from the biofortified purple-fleshed potato test meal is likely to be related to its higher PP content, linked to its purple-fleshed color, compared with the yellow-fleshed color of the regular variety, as shown elsewhere (23). In a series of human iron absorption studies using bean hulls as the source of PP, FIA from a low inhibitory test meal decreased by 45% when 200 mg PP was added (46). Similarly, FIA was reduced from 10.7% to 2.7% at PP GAE amounts of 17 mg and 167 mg, respectively, in iron-biofortified pearl millet test meals (47). Phenolic acids in sweetpotato and potato consist of a mixture that is highest in chlorogenic acids (hydroxycinnamic acid derivatives) followed by rutin (flavonol), caffeic acid (caffeoylquinic acid derivatives), and in purple-fleshed potatoes, anthocyanins (23, 48). In in vitro digestions with boiled sweetpotato and potato, bioaccessible chlorogenic acid was released after gastric and intestinal digestion, which might chelate iron during digestion making it unavailable for absorption (49). Although PPs may impair iron absorption, they also have positive health effects including their antioxidant properties (23, 48, 50). On the other hand, β-carotene in orange-fleshed sweetpotato might prevent the chelating effect of phytate and phenolic acids on iron, improving its absorption (51, 52). Since we did not analyze β-carotene in the orange-fleshed sweetpotato test meals, the potential effect remains unclear.

The AA concentration was low in the biofortified sweetpotato test meal, but in both potato test meals and in the regular sweetpotato test meal the molar ratio of AA:Fe was >1, the threshold for enhancing iron absorption (21, 53). AA is sensitive to food processing and meal preparation. Microwave heating decreased AA content in potatoes by 61–94% (54), and freezing for >5 wk resulted in losses of 23% (55). We found that 54% and 46% of AA was lost in the steamed, frozen, and microwaved regular and biofortified sweetpotato test meals, respectively. In Peru, we found that 17% and 14% of AA was lost during boiling and mashing. In addition, while AA content in the biofortified test meals was stable during storage, AA concentration in the regular potato test meals decreased by >50% during the study.

Calcium content of the regular and biofortified sweetpotato test meals was 114 and 179 mg, respectively, which includes the added calcium through milk (∼34 mg). In contrast, calcium content was only 26 and 37 mg in the regular and biofortified potato test meals. Calcium is a dose-dependent iron inhibitor at amounts >50 mg in a meal (56–58) and the higher calcium content in the sweetpotato meals may have contributed to the lower FIA from those meals. Human intestinal Caco-2 cells exposed to high calcium concentrations show different gene-expression patterns and localization sites for the divalent metal transporter 1 (DMT1) and ferroportin over time, suggesting a possible influence of dietary calcium on key transporters involved in iron absorption (59, 60).

Iron status and inflammation also influence iron absorption (42, 61) through modulation of circulating hepcidin (62). In Peru, 86% of the women had PF ≤25 µg/L and the geometric mean (95% CI) PF concentration was lower (9.7; 7.6, 12.3 µg/L) than in Malawi (21.4; 15.0, 30.5 µg/L), where 58% of the women had SF ≤25 µg/L. Thus, poorer iron status in Peru likely contributed to higher iron absorption compared with Malawi. In contrast, inflammation was similarly low in both settings and is therefore unlikely to have affected iron absorption (63).

Another factor that probably affects iron absorption in Peru is altitude. The Peruvian study population lives in Huancavelica at 3600 m above sea level. In a Bolivian study looking at the effect of living at high altitude, lower body iron measurements were found despite normal Hb values corrected for altitude, suggesting that the women were able to enhance dietary iron absorption to acquire the additional iron needed for RBC mass expansion (64). These findings are in accordance with current studies, which suggest that hypoxia-inducible factor 2 (HIF-2) mechanisms increase intestinal iron absorption by upregulating erythropoietin (EPO) and DMT1 activation and suppressing hepcidin when iron deficiency or increased erythropoiesis occurs (65). High-altitude populations in Tibet show modification of HIF-2–associated genes (66). The influence of high altitude on FIA needs to be investigated in follow-up studies.

Meals based only on potatoes are very common in the Andean highlands. Such a meal containing 500 g of either regular yellow-fleshed or biofortified purple-fleshed potatoes would make a substantial contribution to iron nutrition by providing 33% of the daily requirement for absorbed iron in women of reproductive age (39).

Since de Haan et al. (15) found daily potato intakes of up to 800 g in the region, the daily iron contribution of potato could be even higher. Further, conventionally bred iron-biofortified yellow-fleshed potato varieties currently developed by the CIP are expected to have a higher FIA, because of the yellow variety's lower PP content compared with the biofortified purple-fleshed variety tested in this study, and studies are planned to test these varieties. In contrast, due to lower iron absorption from the orange-fleshed sweetpotato test meals, the contribution to daily iron requirements in women would be 10% from the regular compared with 18% from the biofortified sweetpotato. However, this contribution from the biofortified sweetpotato might increase to 25% when consumed by women with low iron stores (SF ≤25 µg/L). Since habitual consumption data of sweetpotato are not available from Malawi, its contribution to daily iron intake is difficult to estimate. Considering that sweetpotato would likely be consumed in combination with other foods that contain iron enhancers and inhibitors, the daily iron contribution may vary. Several approaches could increase iron absorption from sweetpotato in a mixed Malawian diet, as follows: *1*) sweetpotato AA concentration could be increased by breeding, *2*) preparation methods could be used that do not degrade AA, *3*) AA could be added additionally to the sweetpotato meal (67), and/or *4*) food-to-food fortification could be achieved by adding AA-rich fruits to sweetpotato meals.

To conclude, both of the tested varieties of iron-biofortified sweetpotato and potato could potentially contribute to improved iron intakes and absorption, but breeding for varieties with higher iron, AA, and lower PP concentrations to increase iron bioavailability should be a focus of future research. In addition, iron absorption from biofortified sweetpotatoes and potatoes should be evaluated as a component of a mixed diet in other settings in Africa and Asia. Also, the high iron absorption from potato should be confirmed in populations living at lower altitudes.

## Supplementary Material

nxaa267_Supplemental_File

## Notes

The Malawi study was supported by the International Potato Center's Sweetpotato Action for Security and Health in Africa project, which is funded by the Bill & Melinda Gates Foundation (OPP1019987). The Peru study was funded by USAID and the CGIAR Research Program on Roots, Tubers, and Bananas through the CGIAR Trust Fund Donors (https://www.cgiar.org/funders/).

Author disclosures: The authors report no conflicts of interest.

Supplemental Material is available from the “Supplementary data” link in the online posting of the article and from the same link in the online table of contents available at https://academic.oup.com/jn/.

MBZ and RW are co-senior authors.

Abbreviations: AA, ascorbic acid; CIP, International Potato Center; CRP, C-reactive protein; DMT1, divalent metal transporter 1; GAE, gallic acid equivalents; Hb, hemoglobin; HIF-2, hypoxia-inducible factor 2; ICP-MS, inductively coupled plasma–MS; ID, iron deficiency; IDA, iron deficiency anemia; LMIC, low and middle-income country; PA, phytic acid; PF, plasma ferritin; PP, polyphenol; SF, serum ferritin.
