# Supplementary material for: Iron Absorption from Iron-Biofortified Sweetpotato Is Higher Than Regular Sweetpotato in Malawian Women while Iron Absorption from Regular and Iron-Biofortified Potatoes Is High in Peruvian Women
Source: J Nutr. 2020 Nov 13;150(12):3094–102. doi: 10.1093/jn/nxaa267 (PMC7726126; doi:10.1093/jn/nxaa267)
Supplement: nxaa267_Supplemental_File [file nxaa267_supplemental_file.docx]

**Iron absorption from iron-biofortified sweetpotato is higher than regular sweetpotato in Malawian women while iron absorption from regular and iron-biofortified potatoes is high in Peruvian women Roelinda Jongstra “Online Supplementary Material”**

*Meal preparation sweetpotato study Malawi*

The December 2018 sweetpotato harvest was cured and transported by air and road from Maputo, Mozambique to the study site in Zomba, Malawi in early January 2019. We prepared both the regular and biofortified test meals using the same procedure with the regular test meals first to avoid cross contamination We washed the sweetpotato roots three times under running water to remove soil residues before peeling. Thereafter, we washed them once more and removed black spots before cutting the roots in equal parts of approximately 100 g. Subsequently, we steamed the sweetpotato in 40 kg batches, packed in food grade plastic steaming bags to avoid contamination from the steamer, until all sweetpotato parts were soft (120-150 min.) The steamed sweetpotato pieces were transferred into plastic containers and mashed using an electric meat mincing machine. After cooling down, 400 ± 1 g mashed sweetpotato was portioned into food grade plastic containers and labelled according to sweetpotato variety and steaming batch. Portions were frozen at -20°C. On feeding days, the required number of test meals were thawed using a microwave (900 W, 4 min). We then added 12 g (± 1 g) of butter (Blue Band Original, Unilever Ltd., Nairobi, Kenya), 8 g (± 1 g) of brown sugar (Tseketseke, Illovo, Malawi), 30 mL of UHT processed full cream cow milk (Ultra, Clover, South Africa), and one pinch of iodized salt. We heated each meal portion for another 2 minutes in the microwave (900 W) and stirred manually before serving to the participants.

*Meal preparation potato study Peru*

Both potato varieties were road transported to the research site in Huancavelica, where they were stored until usage. All test meals were prepared fresh every feeding day using the same procedure for both regular and iron-biofortified test meals. The potato tubers were washed under running water and boiled in 6 kg batches completely covered in water for 80 minutes (regular potato) or 120 minutes (biofortified potato) after which the potatoes were peeled and mashed. The mashed potato was kept warm in a water bath (approx. 60°C) until administration to participants. The mashed potato was portioned in two bowls containing 250 g each. The stable isotope solution was administered after the portion in the first bowl was consumed; then the remaining portion provided.
